# Diabetes Mellitus and Its Impact on Patient-Profile and In-Hospital Outcomes in Peripheral Artery Disease

**DOI:** 10.3390/jcm10215033

**Published:** 2021-10-28

**Authors:** Karsten Keller, Volker H. Schmitt, Markus Vosseler, Christoph Brochhausen, Thomas Münzel, Lukas Hobohm, Christine Espinola-Klein

**Affiliations:** 1 Department of Cardiology, Cardiology I, University Medical Center Mainz (Johannes Gutenberg-University Mainz), 55131 Mainz, Germany; Volker.Schmitt@unimedizin-mainz.de (V.H.S.); Vosseler@uni-mainz.de (M.V.); tmuenzel@uni-mainz.de (T.M.); lukas.hobohm@unimedizin-mainz.de (L.H.); espinola@uni-mainz.de (C.E.-K.); 2 Center for Thrombosis and Hemostasis (CTH), University Medical Center Mainz (Johannes Gutenberg-University Mainz), 55131 Mainz, Germany; 3 Medical Clinic VII, Department of Sports Medicine, University Hospital Heidelberg, 69120 Heidelberg, Germany; 4 German Center for Cardiovascular Research (DZHK), Partner Site Rhine Main, 55131 Mainz, Germany; 5 Institute of Pathology, University of Regensburg, 93053 Regensburg, Germany; Christoph.Brochhausen@klinik.uni-regensburg.de

**Keywords:** diabetes mellitus, peripheral artery disease, myocardial infarction, amputation

## Abstract

Background: In patients with peripheral artery disease (PAD), the impact of diabetes mellitus (DM) on patient-profile and adverse in-hospital events is not well investigated. Methods: The German nationwide inpatient sample 2005–2019 was used for this analysis. Hospitalized PAD patients were stratified for DM and the influence of DM on patient-profile and adverse in-hospital events was investigated. Results: Our study comprised 2,654,871 hospitalizations (54.3% aged ≥70 years, 36.7% females) of patients with PAD in Germany 2005–2019. Among these, 864,691 (32.6%) patients had DM and 76,716 (2.9%) died during hospitalization. Diabetic PAD patients revealed an aggravated cardiovascular profile (Charlson Comorbidity Index: 6.0 (5.0–8.0) vs. 4.0 (3.0–5.0), *p* < 0.001). PAD patients with DM showed a higher rate of in-hospital mortality (3.5% vs. 2.6%, *p* < 0.001), as well as major adverse cardiovascular and cerebrovascular events (MACCE, 4.7% vs. 3.3%, *p* < 0.001) and had more often operated with amputation surgery (16.4% vs. 9.1%, *p* < 0.001). DM was an independent predictor of in-hospital mortality (OR 1.077 (95%CI 1.060–1.093), *p* < 0.001) and MACCE (OR 1.118 (95%CI 1.103–1.133), *p* < 0.001). In addition, amputations were also associated with DM (OR 1.804 (95%CI 1.790–1.818)), *p* < 0.001). Conclusions: DM is associated with an unfavorable clinical patient-profile and higher risk for adverse events in PAD patients, including substantially increased in-hospital mortality as well as MACCE rate, and were more often associated with amputation surgeries.

## 1. Introduction

Peripheral artery disease (PAD) is an atherosclerotic disorder of the extremities leading to vascular stenoses/occlusions with consecutive perfusion-undersupply of succeeding tissues [1]. This major cardiovascular disease represents number three of the most common atherosclerotic diseases following coronary artery disease and stroke and is associated with increased morbidity and mortality [2]. PAD is concerned with an elevated risk for cerebrovascular and coronary disease as well as mortality [3]. DM associated lower extremity complications concern approximately 131 million people worldwide with rising tendency [4]. Approximately 20–30% of patients with PAD suffer from diabetes mellitus (DM), which is the second most relevant risk factor for development of PAD after cigarette smoking [5]. On the other hand, diabetics are at more than doubled risk of developing PAD in contrast to the general population [4]. The prevalence of DM was shown to be even higher in PAD compared to patients with coronary artery disease [6]. As the leading metabolic disorder, DM is a worldwide epidemic and represents a well-known relevant risk factor for atherosclerotic diseases [7]. Whereas the prevalence of cigarette smoking is decreasing worldwide, the prevalence of DM is rising steadily [8,9]. Besides the disease severity and duration of DM itself, quality of DM treatment was identified as a crucial factor in the development of PAD [10]. Clinical profile of PAD patients with and without DM significantly differs, with disfavor for diabetics and poor clinical outcome especially in chronic limb-threatening ischemia [11]. DM was associated with a 3.5-fold elevated risk in men and an 8.6-fold elevated risk in women for intermittent claudication [12]. The risk of patients with DM developing PAD was shown to be two- to four-fold increased and aggravated in presence of coronary artery disease [13]. PAD patients with DM have a risk of about 20% for major adverse cardiovascular events in short-term follow-up [14]. Further, PAD patients with DM are more likely to receive major and minor amputations compared to patients without DM [15]. In a recent meta-analysis including more than 58,000 PAD patients from 31 studies, DM was associated with a 67% elevated long-term mortality [16]. While temporal trend analysis revealed a decrease of symptomatic PAD incidence and prevalence in the general population [8], a Canadian study found no decrease of PAD-associated hospitalizations between 2006 and 2019 in people with diabetes [17]. DM is a substantial risk factor for amputation with a 10 to 15 elevated incidence of amputation in patients with DM compared to individuals without DM [18]. In a large study including more than 118,000 patients, DM was shown to be even more strongly associated with amputation than with coronary artery disease [15]. Time trend studies revealing overall decreasing amputation rates in PAD demonstrated constant rates in patients with concomitant DM and even an increased risk for amputation from 10 to 30% in PAD patients with DM and ulcers between 2005 and 2013. Vast differences in amputation risk were seen depending on regional, racial and ethnic, as well as socioeconomic, status [19]. Further, age and higher HbA1c levels were identified as risk factors for amputation [20]. In line with this, a study from Dallas County, USA found a steadily increasing amputation rate in diabetics between 2015 and 2019 with rising numbers of minor amputations but a decrease in major amputations. The reason for this difference was unclear, but authors found that the most possible explanations were improved preventive practices and wound healing, including limb salvage surgeries resulting in limb preservation, which included early intervention with minor amputation leading to lower rates of major amputations [21]. These findings were confirmed by a large Japanese investigation in which the rate of major amputations declined and a stable rate of minor amputations was found between 2013 and 2018. However, incidence of major amputations in patients with DM was 10 times and of minor amputations 15 times higher compared to non-diabetics [18]. Importantly, in diabetics amputation has a vast impact not only on morbidity but also on mortality, since amputation is associated with increased mortality rates, with higher risk for death after major amputation compared to minor amputation [22]. Analyses from Germany on data from public health insurance companies revealed differing findings: a study within the time period 2009–2011 found increasing rates of diabetes in PAD patients and increasing amputation as well as mortality rates especially in more severe cases of PAD measured by the Rutherford categorization, concluding poor outcome, particularly in critical limb ischemia, with less effective performance of angiographies and revascularizations in this patient group [23]. A study of nationwide data in Germany on PAD patients from 2005, 2007, and 2009 confirmed a decrease in major amputation and an increase in minor amputation as well as an increasing mortality rate in patients with claudicatio [24]. A recent analysis on data from a large German insurance company regarding the time period between 2008 and 2016 revealed an increased prevalence of PAD with raising hospitalizations, whereas the number of major amputations decreased which was explained by higher numbers of interventions on older and sicker patients. Interestingly, lower rates of diabetes within PAD patients were found over time in this study [25]. With the present analysis, we aimed to give a recent insight into the association between DM and patient characteristics, adverse in-hospital events and outcome for PAD patients in Germany. For this, all patients hospitalized due to PAD within the time period between 2005 and 2019 in Germany were stratified to DM and no DM and analysed regarding the following parameters: the prevalence of DM in patients with PAD, differences in clinical profile of PAD patients with and without DM, and differences between PAD patients with and without DM concerning amputation, MACCE and mortality. Hence, the present study represents a current comprehensive investigation of hospitalized PAD patients in Germany with regards to diabetic state, including comparison within a period of 15 years.

## 2. Material and Methods

We analysed all hospitalization cases of patients with a main diagnosis of PAD in Germany during the years 2005-2019 (source: RDC of the Federal Statistical Office and the Statistical Offices of the federal states, DRG Statistics 2005–2019, and own calculations). For the present analysis, we included all hospitalized patients with a main diagnosis of PAD (ICD-code I70.2). Patients’ main diagnosis is that which is mainly responsible for patients’ hospitalization (admission to the hospital) [26].

In Germany, diagnoses are coded according to the established coding guidelines ICD-10-GM (International Classification of Diseases, 10th Revision with German Modification) and diagnostical, surgical and interventional procedures with OPS codes (surgery, diagnostic and procedures codes [Operationen und Prozedurenschlüssel]) [27,28] and the Federal Statistical Office of Germany (Statistisches Bundesamt, Wiesbaden, Germany) gathers all data from all inpatient cases in Germany, coded and processed according to the diagnosis related groups (DRG) system. 

In this study, we included all hospitalizations of patients admitted due to PAD, who were identified by the ICD-code I70.2 during the observational period. Within the included hospitalization cases of PAD, patients were stratified for the presence of DM as a secondary diagnosis (ICD-codes E10–E14). We analysed the influence of DM on cardiovascular profile, adverse in-hospital events and amputation rate in PAD patients. 

### 2.1. Study Endpoints and Adverse in-Hospital Events

The primary study outcome was defined as in-hospital death of all-causes. The secondary study outcome comprised major adverse cardiovascular and cerebrovascular events (MACCE, composite of all-cause in-hospital death, acute myocardial infarction (ICD-code I21), and/or ischemic stroke (ICD-code I63)). Further, the prevalence of other adverse events during in-hospital stay such as cardio-pulmonary resuscitation (CPR; OPS-code 8–77), pneumonia (ICD-codes J12–J18), shock (ICD-code R57), pulmonary embolism (ICD-code I26), deep venous thrombosis and/or thrombophlebitis (DVT, ICD-code I80, I81, I82), myocardial infarction (MI, ICD-codes I21, I22), acute kidney injury (AKI, ICD-code N17), stroke (ischemic and hemorrhagic stroke, ICD-codes I61–64), intracerebral bleeding events (ICB, ICD-code I61), gastro-intestinal bleeding (GIB, ICD-codes K920-K922), and transfusion of blood components (OPS code 8–800) were assessed.

#### Definitions

In this study, obesity was defined according to the recommendations of the WHO (World Health Organization) as a body mass index ≥30 kg/m^2^. Shock as well as CPR were defined according to current European guidelines [29,30,31]. Major amputations were defined as amputations above the ankle (OPS-code: 5–864) and minor amputations comprised amputations below the ankle (OPS-code: 5–865). Amputations of the upper extremities and amputations due to reasons other than limb ischemia, such as venous ulceration, trauma and malignancy, were not included in the present analysis [24,32].

### 2.2. Ethical Aspects

In accordance with German law, an approval by an ethical committee as well as informed consent of the patients were not required, since the present study did not involve direct access by the study investigators of data on individual patients.

### 2.3. Statistical Methods

Descriptive statistical comparisons of PAD patients with and without DM were computed as median and interquartile range (IQR) or as absolute numbers and corresponding percentages. Continuous variables were compared with the Mann-Whitney-U test and for categorical variables the Fisher’s exact or the chi^2^ test were used, as appropriate. We used the Bonferroni-Holm method to correct the comparison results for multiple testing.

The investigation of the influence of DM on adverse in-hospital events and in-hospital death in PAD patients was performed using univariable and multivariable logistic regression models given as odds ratio (OR) and 95% CI. The multivariable regression models were adjusted as follows:

- Adjustment I: age, sex, cancer, heart failure, coronary artery disease (CAD), chronic obstructive pulmonary disease, essential arterial hypertension, acute and chronic kidney disease, atrial fibrillation/flutter (AF), and hyperlipidemia.
- Adjustment II: age, sex, cancer, heart failure, CAD, chronic obstructive pulmonary disease, essential arterial hypertension, acute and chronic kidney disease, AF, hyperlipidemia and treatment year.

Statistical significance was presupposed in case of *p*-value < 0.05 (two-sided). Statistical analyses were performed with the software SPSS^®^ (version 20.0; SPSS Inc., Chicago, IL, USA). 

## 3. Results

Our study comprised 2,654,871 hospitalizations (54.3% aged ≥70 years, 36.7% females) of patients with PAD in Germany during the years 2005–2019. Among these, 864,691 (32.6%) patients were coded with DM and 76,716 (2.9%) reached the primary outcome of in-hospital death during hospitalization. 

### 3.1. Clinical Profile of PAD Patients with and without DM

DM type II was found in 96.4% of the PAD patients with DM. A small minority of 2.3% of the PAD patients with DM had a DM type I and in only 1.3% was the subtype unknown or not coded (Table 1).

PAD patients with DM were older (median 73.0 vs. 71.0 years, *p* < 0.001), more often male (65.8% vs. 62.1%, *p* < 0.001) and required a prolonged in-hospital stay (8.0 vs. 6.0 days, *p* < 0.001) compared to non-diabetic patients. We observed an aggravated cardiovascular risk profile in diabetics with a higher prevalence of obesity, hyperlipidemia and arterial hypertension. In line with these findings, PAD patients with DM suffered more often from cardiovascular diseases such as CAD, heart failure and AF (Table 1). As expected, acute and chronic kidney disease were more common in PAD patients with DM. Amputation surgeries were more often performed in PAD patients with DM than in those without (16.4% vs. 9.1%, *p* < 0.001) with a pronounced difference regarding minor amputations (11.8% vs. 5.7%, *p* < 0.001) compared to major amputations (6.1% vs. 4.0%, *p* < 0.001) (Table 1). The Charlson Comorbidity Index revealed a substantially higher value in diabetic than in non-diabetic PAD patients (in median 6.0 (5.0–8.0) vs. 4.0 (3.0–5.0), *p* < 0.001) (Table 1). A higher Charlson comorbidity index was associated with increased in-hospital mortality (OR 1.628 (95%CI 1.619–1.636)), MACCE (OR 1.659 (95%CI 1.652–1.667)), amputation (OR 1.263 (95%CI 1.259–1.266)), minor amputation (OR 1.202 (95%CI 1.199–1.206)), and major amputation rate (OR 1.316 (95%CI 1.310–1.321)) in PAD patients with DM.

### 3.2. Influence of DM on Outcomes in PAD Patients

**Figure 1:**
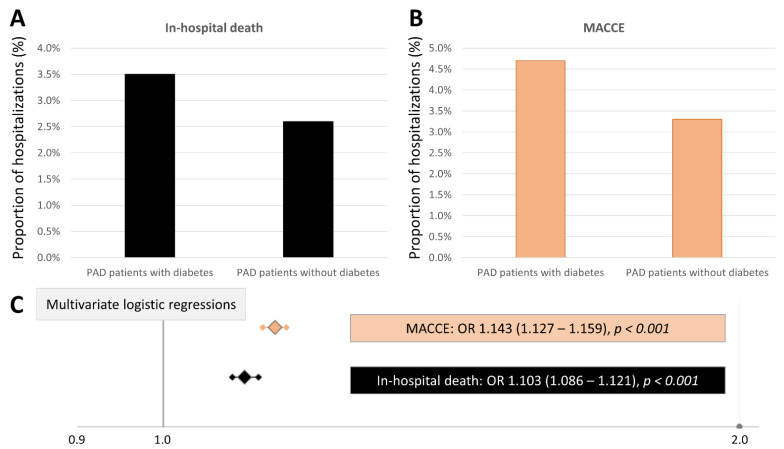
DM affecting in-hospital mortality and MACCE during hospitalizations of PAD patients. (**A**): Comparison of in-hospital mortality of PAD patients stratified for DM. (**B**): Comparison of MACCE rate of PAD patients stratified for DM. (**C**): Multivariate regression models identifying an independent association of DM with in-hospital death as well as MACCE in PAD patients (adjustment II).

Compared to non-diabetics, PAD patients with DM reached the primary outcome of in-hospital death during hospitalization more frequently (3.5% vs. 2.6%, *p* < 0.001) and had higher MACCE rates (4.7% vs. 3.3%, *p* < 0.001) (Table 1, Figure 1). In parallel, the rate of MI and stroke was elevated in diabetics. Additionally, PE and pneumonia occurred more often in diabetic PAD patients, whereas DVT was more frequent in PAD patients without DM. 

While the occurrence of GIB and the necessity of the transfusion of blood constituents was higher in PAD patients with diabetes, prevalence of ICB during in-hospital stay did not differ between groups (Table 1). 

DM was an independent predictor of in-hospital mortality (multivariable logistic regression (adjustment I): OR 1.077 (95%CI 1.060–1.093); multivariable logistic regression (adjustment II): OR 1.103 (95%CI 1.086–1.121)) and MACCE (multivariable logistic regression (adjustment I: OR 1.118 (95%CI 1.103–1.133); multivariable logistic regression (adjustment II): OR 1.143 (95%CI 1.127–1.159)) (Table 2, Figure 1). These results regarding the association between DM and in-hospital death (univariable logistic regression: females: OR 1.358 (95%CI 1.329–1.389); males: OR 1.381 (95%CI 1.354–1.408); multivariable logistic regression (adjustment II): females: OR 1.123 (95%CI 1.097–1.150); males: OR 1.087 (95%CI 1.065–1.110)) as well as DM and MACCE (univariable logistic regression: females: OR 1.451 (95%CI 1.423–1.480); males: OR 1.468 (95%CI 1.443–1.493); multivariable logistic regression (adjustment II): females: OR 1.157 (95%CI 1.133–1.182); males: OR 1.132 (95%CI 1.112–1.153)) were similar in female and male patients. 

Age-dependent analysis, showed an independent association of DM and in-hospital death in the 5th to 9th decade of life (Table 3), whereas DM was associated with MACCE in the 3rd and 5th to 9th decade of life of PAD patients (Table 4).

In line with this finding, DM affected occurrence of MI (OR 1.219 (95%CI 1.186–1.252)) and stroke (OR 1.322 (95%CI 1.275–1.370)). PAD without DM was associated with higher rates of DVT, but did not influence PE development independently (Table 2).

DM was not associated with higher rates of ICB or GIB, but was related to an elevated number of transfusions of blood constituents. 

### 3.3. Influence of DM on Amputation Surgeries in Patients with PAD

Remarkably, DM was strongly associated with amputation surgery (OR 1.804 (95%CI 1.790–1.818)) in PAD patients; especially, minor amputations (OR 2.003 (95%CI 1.984–2.022)), but also major amputations (OR 1.464 (95%CI 1.447–1.482)) were independent of age, sex and comorbidities associated with DM (Table 2). The association of DM with amputation was visible for all PAD patients aged at least 20 years and older (Table 5).

## 4. Discussion

The present study focused on the influence of DM on patient characteristics, outcomes and amputations of more than 2.5 million hospitalizations of PAD patients in Germany 2005–2019. The results of this large nationwide study provided strong evidence regarding an important association of DM with these characteristics and outcomes and the study results can be summarized as follows: 

(I) Approximately one third of the PAD patients had an additional diagnosis of DM, which was predominantly present as type II DM. (II) Patients with PAD and diabetes were older and showed an unbeneficial clinical profile with higher prevalence of cardiovascular risk factors and atherosclerotic comorbidities compared to non-diabetics. (III) Amputations were 1.8-fold more frequently performed in diabetic PAD patients compared to non-diabetics. (IV) In-hospital mortality (1.3-fold) and MACCE rate (1.4-fold) were substantially elevated in patients with additional diagnosis of DM. (V) DM was an independent predictor of in-hospital mortality and MACCE. 

Lower extremity arterial disease is a frequent manifestation of atherosclerosis that is estimated to affect more than 200 million people worldwide, of whom nearby 40 million are living in Europe. In the United States, 8.5 million people above the age of 40 years suffer from PAD, which is associated with significantly increased morbidity, mortality, and quality of life impairment [1,33,34]. The total number of individuals affected by PAD increased by 23% in the last 10 years as a result of population growth, global ageing of the population, increased incidence of DM worldwide and of smoking particularly in low- and middle-income countries [1,5,35]. 

Nevertheless, data regarding a co-prevalence of PAD with DM and the influence of DM on patient-profile, as well as on outcomes in Europe, are scarce [1,36], although this knowledge is relevant for future secondary prevention strategies in this patient collective and might be also important for adequate management of public health and health care service planning [37,38]. Data from several studies suggest a strong interaction between DM and PAD [1,5,35] and DM is one of the key risk factors regarding the development of PAD [5,39]. Since DM is related to a pro-atherosclerotic effect in several vascular beds, DM is accompanied by organ failure in several organs affecting primarily the kidneys, heart, and eyes and is related to poor outcome [39]. In line with previous studies [39], our analysis found that approximately one third of the PAD patients suffered additionally from DM and the large majority of diabetic PAD patients had type II DM. In contrast, other studies reported a lower prevalence of DM in PAD patients [3]. Since patients‘ age, the duration of DM, and peripheral neuropathy are associated with an increased risk of PAD in patients with pre-existing DM [40], the higher prevalence in our study might be attributed to the aging German population. Thus, the high prevalence of DM underlines the outstanding role of DM in PAD. In line with previous studies, PAD patients were predominantly of male sex, although the sex-specific differences were mitigated in the elderly [1]. As previous studies already assumed [7,39], diabetic PAD patients in Germany showed an unfavorable cardiovascular profile with higher frequencies of cardiovascular risk factors and cardiovascular comorbidities. All investigated cardiovascular risk factors including obesity, arterial hypertension and hyperlipidemia were more prevalent in diabetic PAD patients. Most large, population-based studies identified a significant and independent association of arterial hypertension or systolic blood pressure values with PAD [3,41,42,43]. In addition, it is well known, that cardiovascular events are more than twice as likely in patients with DM and arterial hypertension than patients with either disease alone [44]. DM is associated with an increased cardiovascular mortality and arterial hypertension accelerates morbidity and mortality markedly in these patients [45]. In contrast, evidence fails to support a consistent and independent impact of obesity as well as hyperlipidemia on PAD, although the data regarding hyperlipidemia might be influenced by treatment [3]. These findings emphasize the outstanding importance of rigorous and sustained medical treatment in diabetic patients to optimize DM treatment, but also control blood pressure, lower serum cholesterol and reach, and maintain a normal weight [3,44].

Our study demonstrated a substantial 1.8-fold higher risk regarding amputation surgeries in diabetic PAD patients in comparison to non-diabetics during the in-hospital course. In this context, other studies identified male sex, DM and renal disease as independent predictors of amputation [35,46]. This higher risk for amputations in diabetic PAD is of outstanding interest, because it is well known that lower extremity amputation is related to significant morbidity, mortality, and healthcare costs [46]. Regardless of recent advances in PAD treatment and despite overwhelming evidence for reduction of limb loss by revascularization, studies have shown that PAD patients still received an insufficient number of angiographies and revascularizations [23].

The global burden of PAD is strong with a considerable estimated years of life lost in western countries [1]. Although PAD is directly related to increased mortality [1], PAD is additionally accompanied by an elevated risk for cerebrovascular disease and CAD, as well as their acute manifestations of stroke and MI and resulting cardiovascular-related mortality [3]. DM was associated with a statistically significant increased risk of all-cause mortality (OR 1.89 (95%CI 1.51–2.35)) in mid- to long-term follow-up [47]. The results of the above mentioned study are in accordance with our data from Germany, demonstrating a 1.3-fold higher in-hospital death rate as well as a 1.4-fold elevated MACCE rate in diabetics compared to PAD patients without DM. Remarkably, our data demonstrated that DM was an independent and important predictor of in-hospital mortality and MACCE during hospitalization. Since PAD is concerned with an elevated risk for cerebrovascular and coronary disease as well as mortality [3], our study revealed that a co-prevalence of DM in PAD increased rates of MACCE and in-hospital substantially by more than 30%. This outstanding association between DM and outcome recognized in our results is important for adequate management of public health and health care service planning [37,38]. Future studies should elucidate the treatment adherence for PAD as well as for DM in patients with co-prevalence of PAD and DM, since treatment adherence might be a key factor for prevention of adverse events and death. 

## 5. Limitations 

Our analysis has several limitations. First, the analysis is based on ICD and OPS discharge codes of hospitalized patients, which might be affected by under-reporting/under-coding. In this context, the sensitivity and specificity of our identification of inpatients with PAD with and without DM depend on the completeness as well as the accuracy of the ICD-10 coding in the German NIS administrative database [28,48]. In addition, the ICD-10 coding system was not primarily designed to track impact of DM on outcomes of patients hospitalized with PAD and therefore, these data have to be interpreted with caution. Coding practices might vary across hospitals and regions, and financial incentives for hospitals could influence the accuracy of coding [28,48]. Second, detailed baseline data such as concomitant medications, laboratory markers, and echocardiographic parameters were not available. Third, due to the data structure including only the in-hospital stay, follow-up evaluation after discharge is not possible. Furthermore, the database utilized for the current study does not allow any patient-related analysis, which might introduce bias. Fourth, the German nationwide inpatient sample does not provide information on the sequence of the adverse in-hospital events during a patient’s hospitalizations [28,48]. Fifth, because each record in the German nationwide inpatient sample is limited to the duration of the in-hospital stay, we are not able to provide patient-level information regarding hospital readmission or death and adverse events following discharge [28,48]. We tried to encounter potential confounding and choose a widespread large adjustment of the logistic regression models, but we were not able to completely ensure that additional factors might influence and bias the results, although this is unlikely; thus, this epidemiological approach regarding the adjustment was used to prove a widespread independence of DM as an outstanding predictor for case-fatality rate and adverse in-hospital events during hospitalization.

## 6. Conclusions

(I) Approximately one third of the PAD patients had an additional diagnosis of DM, which was predominantly present as type II DM.

(II) Patients with PAD and diabetes were older and showed an unbeneficial clinical profile with higher prevalence of cardiovascular risk factors and atherosclerotic comorbidities compared to non-diabetics.

(III) Amputations were 1.8-fold more frequently performed in diabetic PAD patients compared to non-diabetics.

(IV) In-hospital mortality (1.3-fold) and MACCE rate (1.4-fold) were substantially elevated in patients with additional diagnosis of DM.

(V) DM was an independent predictor of in-hospital mortality and MACCE. 

## Figures and Tables

**Table 1:** Patients’ characteristics, medical history, presentation and outcome of the included 2,654,871 PAD patients stratified according to the presence of diabetes mellitus.

Parameters	PAD Patients with Diabetes (*n* = 864,691; 32.6%)	PAD Patients without Diabetes (*n* = 1,790,180; 67.4%)	*p*-Value ^†^
Age	73.0 (65.0–79.0)	71.0 (62.0–79.0)	<0.001
Age ≥ 70 years	512,438 (59.3%)	928,893 (51.9%)	<0.001
Female sex *	296,102 (34.2%)	679,226 (37.9%)	<0.001
In-hospital stay (days)	8.0 (3.0–16.0)	6.0 (2.0–13.0)	<0.001
**Diabetes mellitus subtypes**			
Diabetes mellitus type I	19,799 (2.3%)		
Diabetes mellitus type II	833,780 (96.4%)		
Unknown/uncoded diabetes subtype	11,112 (1.3%)		
**Traditional cardiovascular risk factors**			
Obesity	103,253 (11.9%)	98,913 (5.5%)	<0.001
Essential arterial hypertension	596,842 (69.0%)	1,083,759 (60.5%)	<0.001
Hyperlipidaemia	331,467 (38.3%)	594,525 (33.2%)	<0.001
**Comorbidities**			
Cancer	14,523 (1.7%)	32,814 (1.8%)	<0.001
Coronary artery disease	308,501 (35.7%)	421,655 (23.6%)	<0.001
Heart failure	143,658 (16.6%)	187,766 (10.5%)	<0.001
Atrial fibrillation/flutter	161,814 (18.7%)	235,373 (13.1%)	<0.001
Chronic obstructive pulmonary disease	76,858 (8.9%)	166,201 (9.3%)	<0.001
Acute and chronic kidney disease	308,985 (35.7%)	363,763 (20.3%)	<0.001
Anaemia	148,485 (17.2%)	234,460 (13.1%)	<0.001
Charlson comorbidity index	6.0 (5.0–8.0)	4.0 (3.0–5.0)	<0.001
**Amputation treatment**			
Amputation	141,742 (16.4%)	163,099 (9.1%)	<0.001
Minor amputation	101,681 (11.8%)	101,271 (5.7%)	<0.001
Major amputation	52,365 (6.1%)	71,067 (4.0%)	<0.001
**Adverse events during hospitalization**			
In-hospital death	30,129 (3.5%)	46,587 (2.6%)	<0.001
MACCE	40,760 (4.7%)	59,327 (3.3%)	<0.001
Cardio-pulmonary resuscitation	7416 (0.9%)	9221 (0.5%)	<0.001
Shock	7610 (0.9%)	11,884 (0.7%)	<0.001
Myocardial infarction	10,685 (1.2%)	12,082 (0.7%)	<0.001
Pulmonary embolism	1,151 (0.13%)	2,053 (0.11%)	<0.001
Deep venous thrombosis or thrombophlebitis	4767 (0.55%)	10,561 (0.59%)	<0.001
Pneumonia	19,305 (2.2%)	28,103 (1.6%)	<0.001
Acute kidney injury	21,617 (2.5%)	27,023 (1.5%)	<0.001
Stroke (ischaemic or haemorrhagic)	5499 (0.6%)	7621 (0.4%)	<0.001
Intracerebral bleeding	545 (0.03%)	277 (0.03%)	0.504
Gastro-intestinal bleeding	4066 (0.5%)	7091 (0.4%)	<0.001
Transfusion of blood constituents	107,710 (12.5%)	178,379 (10.0%)	<0.001

* Information available for 2,654,741 patients. ^†^ After correction of the *p*-values for multiple testing by the Bonferroni-Holm method *p*-value < 0.001 were corrected to *p* = 0.031.

**Table 2:** Impact of diabetes mellitus on the different adverse in-hospital events in PAD patients (univariable and multivariable logistic regression models).

	Univariable Regression Model		Multivariable Regression Model (Adjustment I)		Multivariable Regression Model (Adjustment II)	
	OR (95% CI)	*p*-Value	OR (95% CI)	*p*-Value	OR (95% CI)	*p*-Value
In-hospital death	1.351 (1.331–1.371)	<0.001	1.077 (1.060–1.093)	<0.001	1.103 (1.086–1.121)	<0.001
Cardio-pulmonary resuscitation	1.671 (1.620–1.723)	<0.001	1.189 (1.152–1.228)	<0.001	1.203 (1.165–1.242)	<0.001
MACCE	1.443 (1.425–1.462)	<0.001	1.118 (1.103–1.133)	<0.001	1.143 (1.127–1.159)	<0.001
Pulmonary embolism	1.161 (1.080–1.248)	<0.001	1.011 (0.939–1.089)	0.772	0.995 (0.924–1.073)	0.905
Pneumonia	1.432 (1.406–1.459)	<0.001	1.106 (1.085–1.127)	<0.001	1.123 (1.101–1.145)	<0.001
Deep venous thrombosis or thrombophlebitis	0.934 (0.903–0.967)	<0.001	0.896 (0.865–0.929)	<0.001	0.887 (0.856–0.919)	<0.001
Acute kidney injury	1.673 (1.643–1.703)	<0.001	–	–	–	–
Myocardial infarction	1.841 (1.794–1.890)	<0.001	1.219 (1.186–1.252)	<0.001	1.241 (1.208–1.276)	<0.001
Shock	1.329 (1.291–1.368)	<0.001	0.950 (0.922–0.979)	0.001	0.954 (0.926–0.983)	0.002
Stroke (ischemic or hemorrhagic)	1.497 (1.446–1.550)	<0.001	1.322 (1.275–1.370)	<0.001	1.338 (1.291–1.387)	<0.001
Intracerebral bleeding	1.052 (0.911–1.216)	0.490	0.913 (0.787–1.058)	0.227	0.920 (0.792–1.067)	0.270
Gastro-intestinal bleeding	1.188 (1.143–1.235)	<0.001	0.957 (0.920–0.996)	0.032	0.971 (0.933–1.010)	0.143
Transfusion of blood constituents	1.286 (1.275–1.296)	<0.001	1.089 (1.080–1.098)	<0.001	1.110 (1.100–1.119)	<0.001
**Amputation treatment**						
Amputation	1.956 (1.941–1.971)	<0.001	1.804 (1.790–1.818)	<0.001	1.822 (1.808–1.837)	<0.001
Minor amputation	2.222 (2.202–2.243)	<0.001	2.003 (1.984–2.022)	<0.001	2.008 (1.989–2.027)	<0.001
Major amputation	1.559 (1.541–1.578)	<0.001	1.464 (1.447–1.482)	<0.001	1.494 (1.476–1.512)	<0.001

**Table 3:** Impact of diabetes mellitus on in-hospital death in PAD patients (univariable and multivariable logistic regression models).

	Univariable Regression Model		Multivariable Regression Model (Adjustment II)	
	OR (95% CI)	*p*-Value	OR (95% CI)	*p*-Value
30–39 years	5.602 (2.422–12.957)	<0.001	1.458 (0.533–3.989)	0.462
40–49 years	2.970 (2.363–3.732)	<0.001	1.635 (1.265–2.112)	<0.001
50–59 years	1.878 (1.735–2.033)	<0.001	1.130 (1.036–1.233)	0.006
60–69 years	1.535 (1.475–1.599)	<0.001	1.070 (1.024–1.118)	0.002
70–79 years	1.409 (1.373–1.446)	<0.001	1.092 (1.063–1.123)	<0.001
80–89 years	1.173 (1.146–1.201)	<0.001	1.086 (1.059–1.113)	<0.001
≥90 years	1.020 (0.971–1.071)	0.435	1.023 (0.973–1.076)	0.374

**Table 4:** Impact of diabetes mellitus on MACCE in PAD patients (univariable and multivariable logistic regression models).

	Univariable Regression Model		Multivariable Regression Model (Adjustment II)	
	OR (95% CI)	*p*-Value	OR (95% CI)	*p*-Value
20–29 years	18.043 (2.485–130.988)	0.004	15.955 (1.074–236.997)	0.044
30–39 years	3.166 (1.598–6.271)	0.001	0.997 (0.431–2.306)	0.994
40–49 years	2.463 (2.078–2.920)	<0.001	1.387 (1.147–1.676)	0.001
50–59 years	1.954 (1.838–2.077)	<0.001	1.206 (1.128–1.290)	<0.001
60–69 years	1.638 (1.585–1.692)	<0.001	1.149 (1.109–1.190)	<0.001
70–79 years	1.491 (1.458–1.524)	<0.001	1.144 (1.118–1.172)	<0.001
80–89 years	1.228 (1.202–1.255)	<0.001	1.114 (1.089–1.140)	<0.001
≥90 years	1.035 (0.987–1.085)	0.152	1.026 (0.977–1.077)	0.305

**Table 5:** Influence of diabetes mellitus on amputation in PAD patients (univariable and multivariable logistic regression models).

	Univariable Regression Model		Multivariable Regression Model (Adjustment II)	
	OR (95% CI)	*p*-Value	OR (95% CI)	*p*-Value
20–29 years	2.736 (1.310–5.717)	0.007	2.483 (1.097–5.623)	0.029
30–39 years	1.643 (1.340–2.014)	<0.001	1.308 (1.040–1.644)	0.022
40–49 years	2.800 (2.629–2.982)	<0.001	2.558 (2.388–2.739)	<0.001
50–59 years	2.799 (2.726–2.875)	<0.001	2.595 (2.522–2.670)	<0.001
60–69 years	2.317 (2.278–2.356)	<0.001	2.122 (2.084–2.160)	<0.001
70–79 years	1.992 (1.966–2.018)	<0.001	1.839 (1.814–1.864)	<0.001
80–89 years	1.524 (1.502–1.547)	<0.001	1.535 (1.512–1.558)	<0.001
≥90 years	1.254 (1.211–1.298)	<0.001	1.301 (1.256–1.347)	<0.001

## Data Availability

The data were provided from the Federal Statistical Office of Germany (Statistisches Bundesamt, DEStatis) (source: RDC of the Federal Statistical Office and the Statistical Offices of the federal states, DRG Statistics 2005–2019, and own calculations).

## Abbreviations

AKI	Acute kidney injury
AF	Atrial fibrillation/flutter
CAD	Coronary artery disease
CI	Confidence interval
CPR	Cardiopulmonary resuscitation
DM	Diabetes mellitus
DRG	Diagnosis Related Groups
DVT	Deep venous thrombosis or thrombophlebitis of the leg veins
GIB	Gastro-intestinal bleeding
ICB	Intracerebral bleeding events
ICD	International Classification of Diseases and Related Health Problems
IQR	Inter-quartile range
OPS	Diagnostic, surgery and procedures codes (Operationen und Prozedurenschlüssel)
OR	Odds ratio
MACCE	Major adverse cardiovascular and cerebrovascular events
PAD	Peripheral artery disease
PE	Pulmonary embolism
RDC	Research Data Center
vs.	Versus
